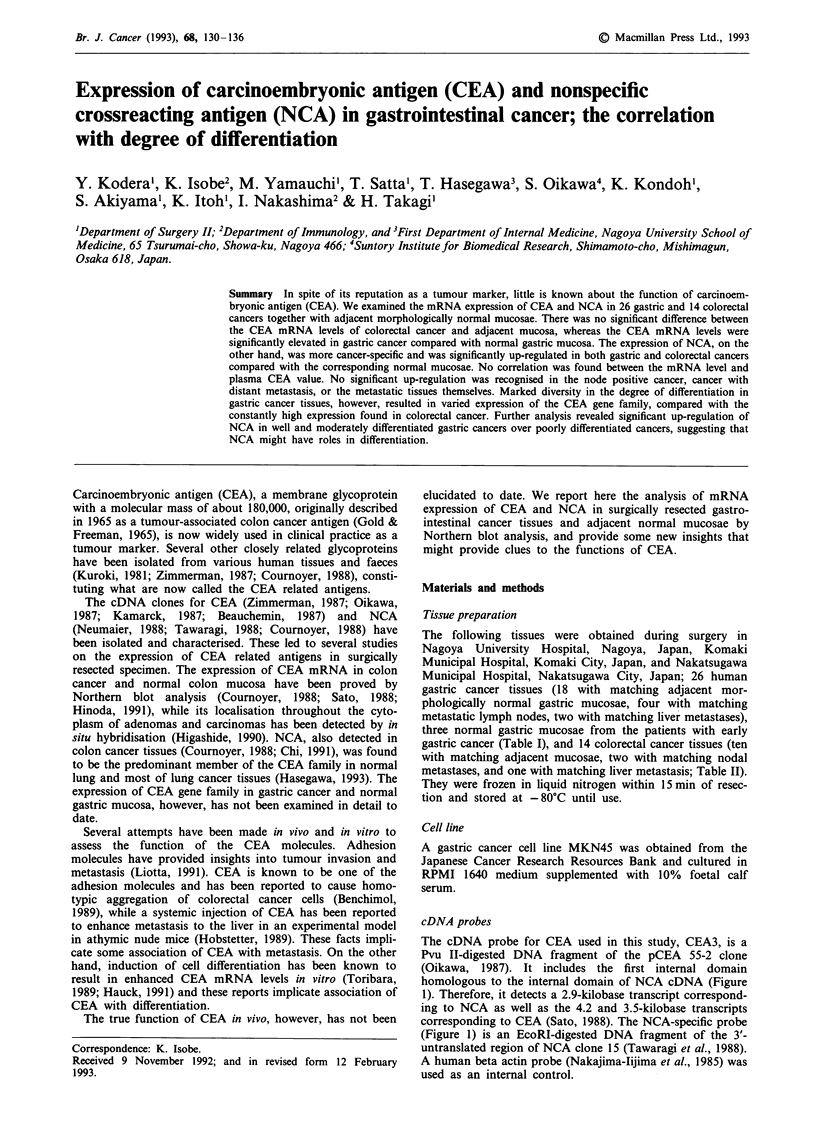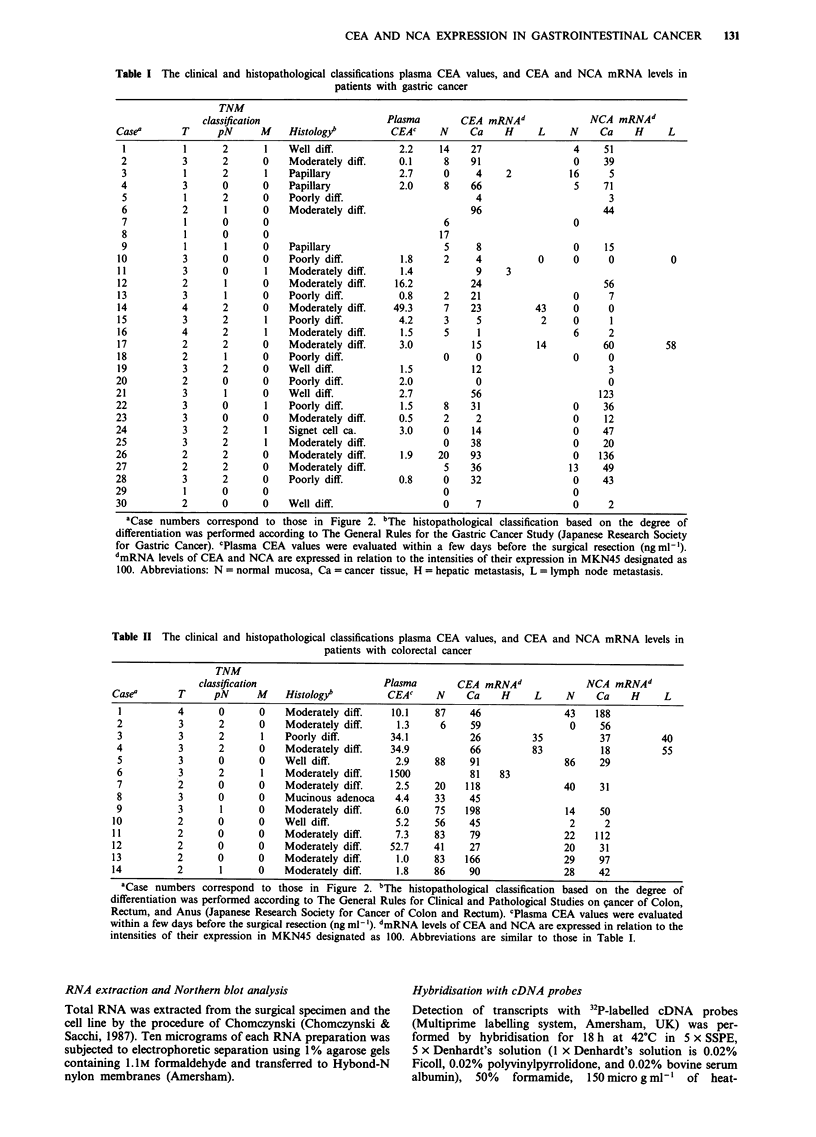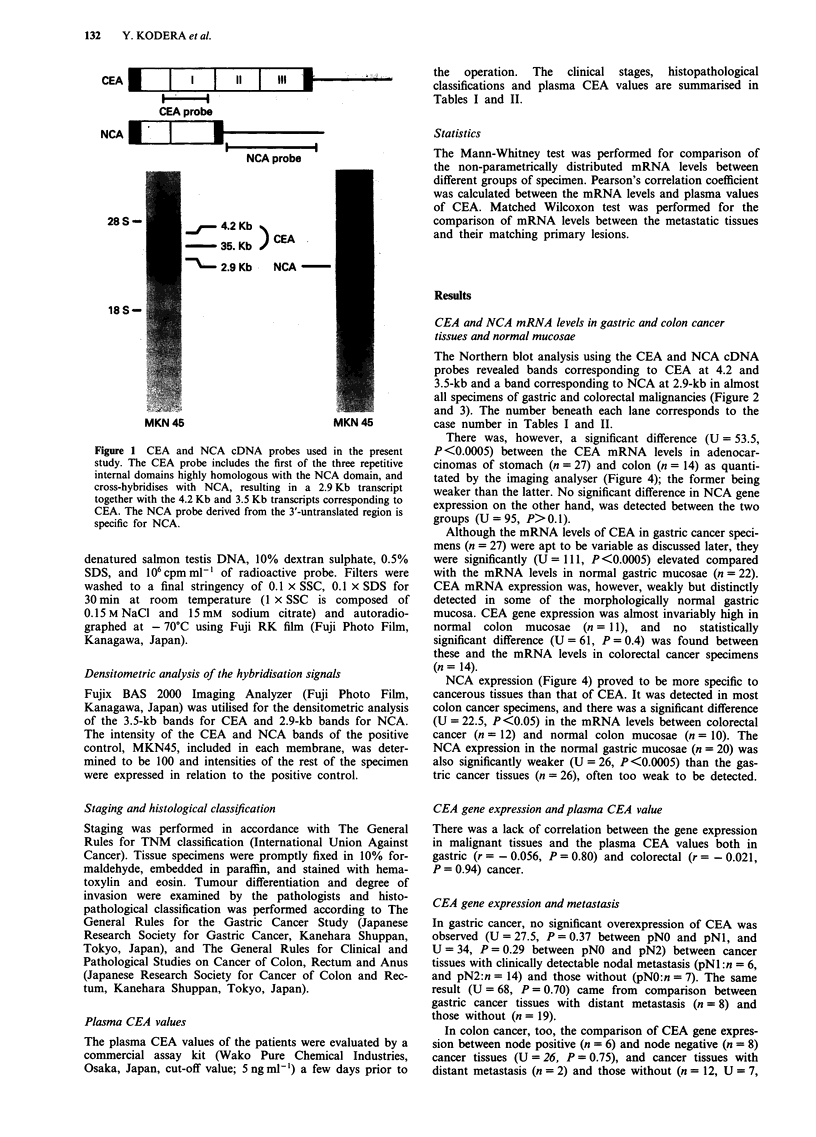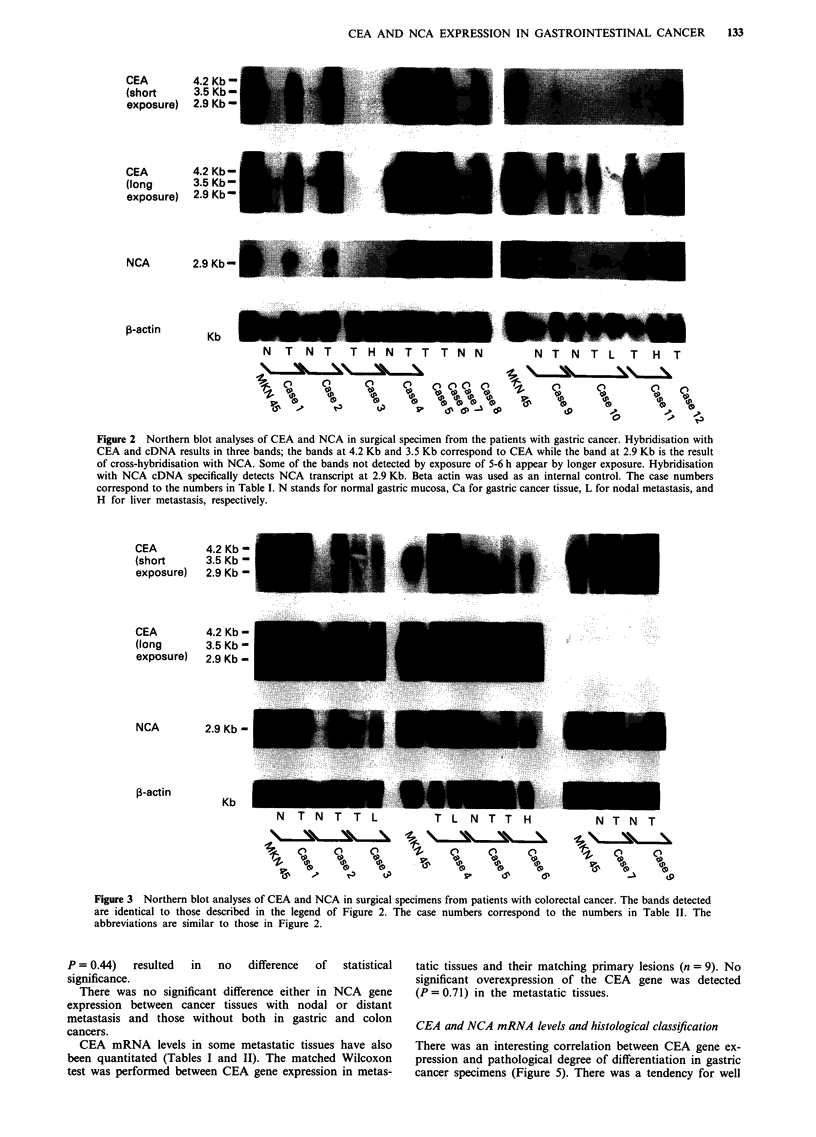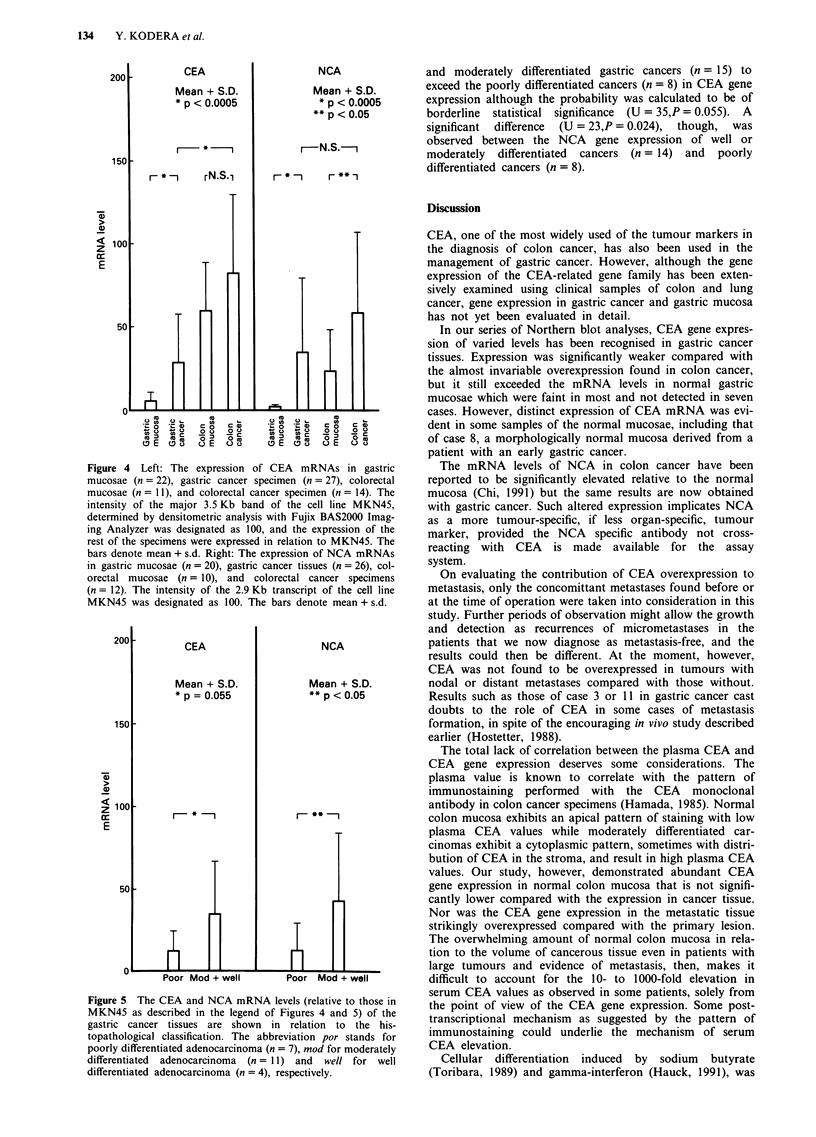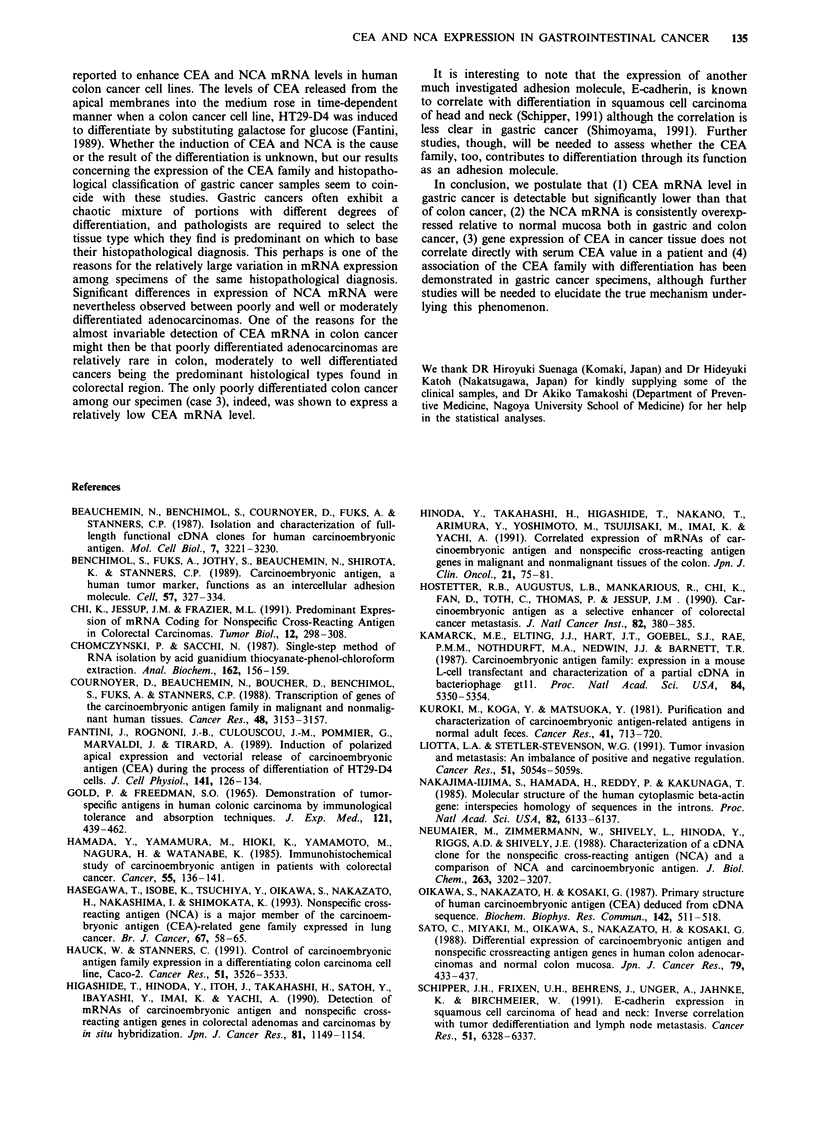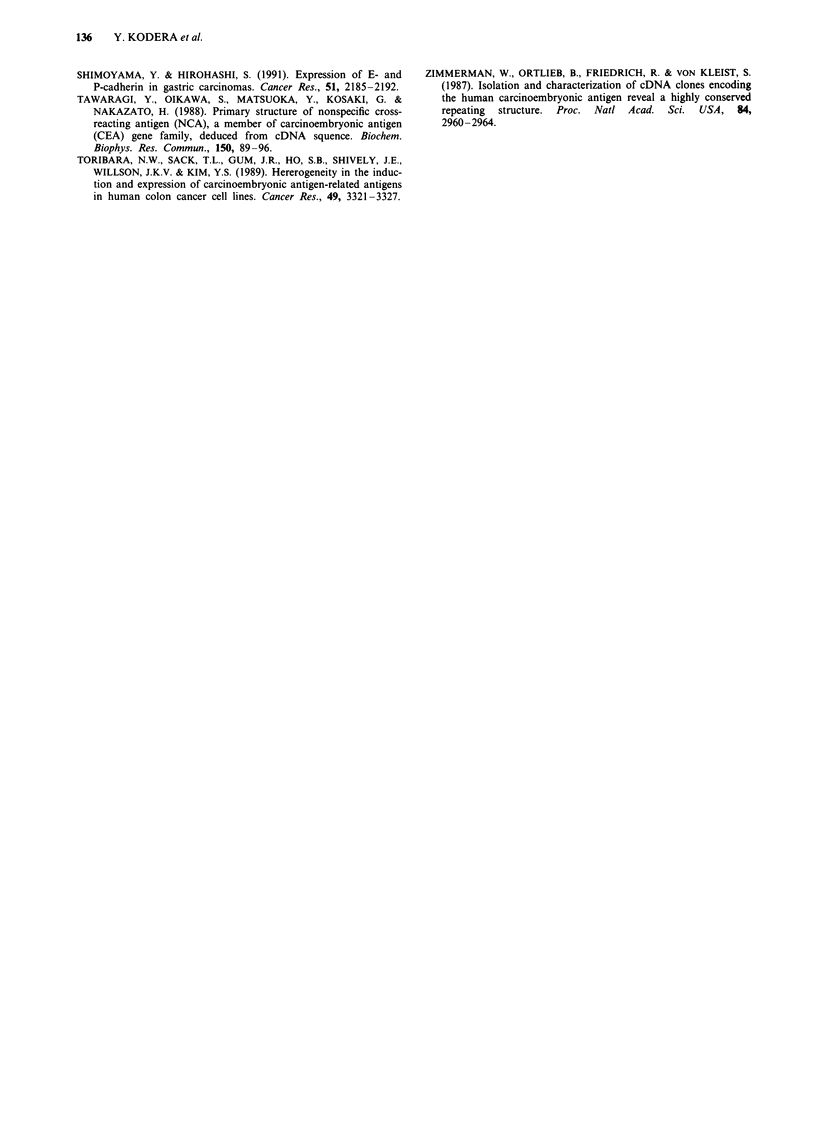# Expression of carcinoembryonic antigen (CEA) and nonspecific crossreacting antigen (NCA) in gastrointestinal cancer; the correlation with degree of differentiation.

**DOI:** 10.1038/bjc.1993.300

**Published:** 1993-07

**Authors:** Y. Kodera, K. Isobe, M. Yamauchi, T. Satta, T. Hasegawa, S. Oikawa, K. Kondoh, S. Akiyama, K. Itoh, I. Nakashima

**Affiliations:** Department of Surgery II, Nagoya University School of Medicine, Japan.

## Abstract

**Images:**


Br. J. Cancer (1993), 68, 130-136 © Macmillan Press Ltd., 1993

### Expression of carcinoembryonic antigen (CEA) and nonspecific crossreacting antigen (NCA) in gastrointestinal cancer; the correlation with degree of differentiation

Y. Kodera[1], K. Isobe[2], M. Yamauchi[1], T. Satta[1], T. Hasegawa[3], S. Oikawa[4], K. Kondoh[1], S. Akiyama[1], K. Itoh[1], I. Nakashima[2] & H. Takagi[1]

[1]*Department of Surgery II;* [2]*Department of Immunology, and* [3]*First Department of Internal Medicine, Nagoya University School of Medicine, 65 Tsurumai-cho, Showa-ku, Nagoya 466;* [4]*Suntory Institute for Biomedical Research, Shimamoto-cho, Mishimagun, Osaka 618, Japan.*

**Summary** In spite of its reputation as a tumour marker, little is known about the function of carcinoembryonic antigen (CEA). We examined the mRNA expression of CEA and NCA in 26 gastric and 14 colorectal cancers together with adjacent morphologically normal mucosae. There was no significant difference between the CEA mRNA levels of colorectal cancer and adjacent mucosa, whereas the CEA mRNA levels were significantly elevated in gastric cancer compared with normal gastric mucosa. The expression of NCA, on the other hand, was more cancer-specific and was significantly up-regulated in both gastric and colorectal cancers compared with the corresponding normal mucosae. No correlation was found between the mRNA level and plasma CEA value. No significant up-regulation was recognised in the node positive cancer, cancer with distant metastasis, or the metastatic tissues themselves. Marked diversity in the degree of differentiation in gastric cancer tissues, however, resulted in varied expression of the CEA gene family, compared with the constantly high expression found in colorectal cancer. Further analysis revealed significant up-regulation of NCA in well and moderately differentiated gastric cancers over poorly differentiated cancers, suggesting that NCA might have roles in differentiation.

Carcinoembryonic antigen (CEA), a membrane glycoprotein with a molecular mass of about 180,000, originally described in 1965 as a tumour-associated colon cancer antigen (Gold & Freeman, 1965), is now widely used in clinical practice as a tumour marker. Several other closely related glycoproteins have been isolated from various human tissues and faeces (Kuroki, 1981; Zimmerman, 1987; Cournoyer, 1988), constituting what are now called the CEA related antigens.

The cDNA clones for CEA (Zimmerman, 1987; Oikawa, 1987; Kamarck, 1987; Beauchemin, 1987) and NCA (Neumaier, 1988; Tawaragi, 1988; Cournoyer, 1988) have been isolated and characterised. These led to several studies on the expression of CEA related antigens in surgically resected specimen. The expression of CEA mRNA in colon cancer and normal colon mucosa have been proved by Northern blot analysis (Cournoyer, 1988; Sato, 1988; Hinoda, 1991), while its localisation throughout the cytoplasm of adenomas and carcinomas has been detected by *in situ* hybridisation (Higashide, 1990). NCA, also detected in colon cancer tissues (Cournoyer, 1988; Chi, 1991), was found to be the predominant member of the CEA family in normal lung and most of lung cancer tissues (Hasegawa, 1993). The expression of CEA gene family in gastric cancer and normal gastric mucosa, however, has not been examined in detail to date.

Several attempts have been made *in vivo* and *in vitro* to assess the function of the CEA molecules. Adhesion molecules have provided insights into tumour invasion and metastasis (Liotta, 1991). CEA is known to be one of the adhesion molecules and has been reported to cause homotypic aggregation of colorectal cancer cells (Benchimol, 1989), while a systemic injection of CEA has been reported to enhance metastasis to the liver in an experimental model in athymic nude mice (Hobstetter, 1989). These facts implicate some association of CEA with metastasis. On the other hand, induction of cell differentiation has been known to result in enhanced CEA mRNA levels *in vitro* (Toribara, 1989; Hauck, 1991) and these reports implicate association of CEA with differentiation.

The true function of CEA *in vivo*, however, has not been elucidated to date. We report here the analysis of mRNA expression of CEA and NCA in surgically resected gastrointestinal cancer tissues and adjacent normal mucosae by Northern blot analysis, and provide some new insights that might provide clues to the functions of CEA.

### Materials and methods

### *Tissue preparation*

The following tissues were obtained during surgery in Nagoya University Hospital, Nagoya, Japan, Komaki Municipal Hospital, Komaki City, Japan, and Nakatsugawa Municipal Hospital, Nakatsugawa City, Japan; 26 human gastric cancer tissues (18 with matching adjacent morphologically normal gastric mucosae, four with matching metastatic lymph nodes, two with matching liver metastases), three normal gastric mucosae from the patients with early gastric cancer (Table I), and 14 colorectal cancer tissues (ten with matching adjacent mucosae, two with matching nodal metastases, and one with matching liver metastasis; Table II). They were frozen in liquid nitrogen within 15 min of resection and stored at −80°C until use.

### *Cell line*

A gastric cancer cell line MKN45 was obtained from the Japanese Cancer Research Resources Bank and cultured in RPMI 1640 medium supplemented with 10% foetal calf serum.

### *cDNA probes*

The cDNA probe for CEA used in this study, CEA3, is a Pvu II-digested DNA fragment of the pCEA 55-2 clone (Oikawa, 1987). It includes the first internal domain homologous to the internal domain of NCA cDNA (Figure 1). Therefore, it detects a 2.9-kilobase transcript corresponding to NCA as well as the 4.2 and 3.5-kilobase transcripts corresponding to CEA (Sato, 1988). The NCA-specific probe (Figure 1) is an EcoRI-digested DNA fragment of the 3′-untranslated region of NCA clone 15 (Tawaragi *et al.*, 1988). A human beta actin probe (Nakajima-Iijima *et al.*, 1985) was used as an internal control.

Correspondence: K. Isobe.
Received 9 November 1992; and in revised form 12 February 1993.

**Table I** The clinical and histopathological classifications plasma CEA values, and CEA and NCA mRNA levels in patients with gastric cancer

| Case[a] | TNM classification T | pN | M | Histology[b] | Plasma CEA[c] | CEA mRNA[d] N | Ca | H | L | NCA mRNA[d] N | Ca | H | L |
|---|---|---|---|---|---|---|---|---|---|---|---|---|---|
| 1 | 1 | 2 | 1 | Well diff. | 2.2 | 14 | 27 | | | 4 | 51 | | |
| 2 | 3 | 2 | 0 | Moderately diff. | 0.1 | 8 | 91 | | | 0 | 39 | | |
| 3 | 1 | 2 | 1 | Papillary | 2.7 | 0 | 4 | 2 | | 16 | 5 | | |
| 4 | 3 | 0 | 0 | Papillary | 2.0 | 8 | 66 | | | 5 | 71 | | |
| 5 | 1 | 2 | 0 | Poorly diff. | | | 4 | | | | 3 | | |
| 6 | 2 | 1 | 0 | Moderately diff. | | | 96 | | | | 44 | | |
| 7 | 1 | 0 | 0 | | | 6 | | | | 0 | | | |
| 8 | 1 | 0 | 0 | | | 17 | | | | | | | |
| 9 | 1 | 1 | 0 | Papillary | | 5 | 8 | | | 0 | 15 | | |
| 10 | 3 | 0 | 0 | Poorly diff. | 1.8 | 2 | 4 | | 0 | 0 | 0 | | 0 |
| 11 | 3 | 0 | 1 | Moderately diff. | 1.4 | | 9 | 3 | | | | | |
| 12 | 2 | 1 | 0 | Moderately diff. | 16.2 | | 24 | | | | 56 | | |
| 13 | 3 | 1 | 0 | Poorly diff. | 0.8 | 2 | 21 | | | 0 | 7 | | |
| 14 | 4 | 2 | 0 | Moderately diff. | 49.3 | 7 | 23 | | 43 | 0 | 0 | | |
| 15 | 3 | 2 | 1 | Poorly diff. | 4.2 | 3 | 5 | | 2 | 0 | 1 | | |
| 16 | 4 | 2 | 1 | Moderately diff. | 1.5 | 5 | 1 | | | 6 | 2 | | |
| 17 | 2 | 2 | 0 | Moderately diff. | 3.0 | | 15 | | 14 | | 60 | | 58 |
| 18 | 2 | 1 | 0 | Poorly diff. | | 0 | 0 | | | 0 | 0 | | |
| 19 | 3 | 2 | 0 | Well diff. | 1.5 | | 12 | | | | 3 | | |
| 20 | 2 | 0 | 0 | Poorly diff. | 2.0 | | 0 | | | | 0 | | |
| 21 | 3 | 1 | 0 | Well diff. | 2.7 | | 56 | | | | 123 | | |
| 22 | 3 | 0 | 1 | Poorly diff. | 1.5 | 8 | 31 | | | 0 | 36 | | |
| 23 | 3 | 0 | 0 | Moderately diff. | 0.5 | 2 | 2 | | | 0 | 12 | | |
| 24 | 3 | 2 | 1 | Signet cell ca. | 3.0 | 0 | 14 | | | 0 | 47 | | |
| 25 | 3 | 2 | 1 | Moderately diff. | | 0 | 38 | | | 0 | 20 | | |
| 26 | 2 | 2 | 0 | Moderately diff. | 1.9 | 20 | 93 | | | 0 | 136 | | |
| 27 | 2 | 2 | 0 | Moderately diff. | | 5 | 36 | | | 13 | 49 | | |
| 28 | 3 | 2 | 0 | Poorly diff. | 0.8 | 0 | 32 | | | 0 | 43 | | |
| 29 | 1 | 0 | 0 | | | 0 | | | | 0 | | | |
| 30 | 2 | 0 | 0 | Well diff. | | 0 | 7 | | | 0 | 2 | | |

[a]Case numbers correspond to those in Figure 2. [b]The histopathological classification based on the degree of differentiation was performed according to The General Rules for the Gastric Cancer Study (Japanese Research Society for Gastric Cancer). [c]Plasma CEA values were evaluated within a few days before the surgical resection ($ng\,ml^{-1}$). [d]mRNA levels of CEA and NCA are expressed in relation to the intensities of their expression in MKN45 designated as 100. Abbreviations: N = normal mucosa, Ca = cancer tissue, H = hepatic metastasis, L = lymph node metastasis.

**Table II** The clinical and histopathological classifications plasma CEA values, and CEA and NCA mRNA levels in patients with colorectal cancer

| Case[a] | TNM classification T | pN | M | Histology[b] | Plasma CEA[c] | CEA mRNA[d] N | Ca | H | L | NCA mRNA[d] N | Ca | H | L |
|---|---|---|---|---|---|---|---|---|---|---|---|---|---|
| 1 | 4 | 0 | 0 | Moderately diff. | 10.1 | 87 | 46 | | | 43 | 188 | | |
| 2 | 3 | 2 | 0 | Moderately diff. | 1.3 | 6 | 59 | | | 0 | 56 | | |
| 3 | 3 | 2 | 1 | Poorly diff. | 34.1 | | 26 | | 35 | | 37 | | 40 |
| 4 | 3 | 2 | 0 | Moderately diff. | 34.9 | | 66 | | 83 | | 18 | | 55 |
| 5 | 3 | 0 | 0 | Well diff. | 2.9 | 88 | 91 | | | 86 | 29 | | |
| 6 | 3 | 2 | 1 | Moderately diff. | 1500 | | 81 | 83 | | | | | |
| 7 | 2 | 0 | 0 | Moderately diff. | 2.5 | 20 | 118 | | | 40 | 31 | | |
| 8 | 3 | 0 | 0 | Mucinous adenoca | 4.4 | 33 | 45 | | | | | | |
| 9 | 3 | 1 | 0 | Moderately diff. | 6.0 | 75 | 198 | | | 14 | 50 | | |
| 10 | 2 | 0 | 0 | Well diff. | 5.2 | 56 | 45 | | | 2 | 2 | | |
| 11 | 2 | 0 | 0 | Moderately diff. | 7.3 | 83 | 79 | | | 22 | 112 | | |
| 12 | 2 | 0 | 0 | Moderately diff. | 52.7 | 41 | 27 | | | 20 | 31 | | |
| 13 | 2 | 0 | 0 | Moderately diff. | 1.0 | 83 | 166 | | | 29 | 97 | | |
| 14 | 2 | 1 | 0 | Moderately diff. | 1.8 | 86 | 90 | | | 28 | 42 | | |

[a]Case numbers correspond to those in Figure 2. [b]The histopathological classification based on the degree of differentiation was performed according to The General Rules for Clinical and Pathological Studies on çancer of Colon, Rectum, and Anus (Japanese Research Society for Cancer of Colon and Rectum). [c]Plasma CEA values were evaluated within a few days before the surgical resection ($ng\,ml^{-1}$). [d]mRNA levels of CEA and NCA are expressed in relation to the intensities of their expression in MKN45 designated as 100. Abbreviations are similar to those in Table I.

*RNA extraction and Northern blot analysis*

Total RNA was extracted from the surgical specimen and the cell line by the procedure of Chomczynski (Chomczynski & Sacchi, 1987). Ten micrograms of each RNA preparation was subjected to electrophoretic separation using 1% agarose gels containing 1.1M formaldehyde and transferred to Hybond-N nylon membranes (Amersham).

*Hybridisation with cDNA probes*

Detection of transcripts with $^{32}P$-labelled cDNA probes (Multiprime labelling system, Amersham, UK) was performed by hybridisation for 18 h at 42°C in 5 × SSPE, 5 × Denhardt's solution (1 × Denhardt's solution is 0.02% Ficoll, 0.02% polyvinylpyrrolidone, and 0.02% bovine serum albumin), 50% formamide, 150 micro g $ml^{-1}$ of heat-

**Figure 1** CEA and NCA cDNA probes used in the present study. The CEA probe includes the first of the three repetitive internal domains highly homologous with the NCA domain, and cross-hybridises with NCA, resulting in a 2.9 Kb transcript together with the 4.2 Kb and 3.5 Kb transcripts corresponding to CEA. The NCA probe derived from the 3'-untranslated region is specific for NCA.

denatured salmon testis DNA, 10% dextran sulphate, 0.5% SDS, and $10^6$ cpm $ml^{-1}$ of radioactive probe. Filters were washed to a final stringency of 0.1 × SSC, 0.1 × SDS for 30 min at room temperature (1 × SSC is composed of 0.15 M NaCl and 15 mM sodium citrate) and autoradiographed at − 70°C using Fuji RK film (Fuji Photo Film, Kanagawa, Japan).

*Densitometric analysis of the hybridisation signals*

Fujix BAS 2000 Imaging Analyzer (Fuji Photo Film, Kanagawa, Japan) was utilised for the densitometric analysis of the 3.5-kb bands for CEA and 2.9-kb bands for NCA. The intensity of the CEA and NCA bands of the positive control, MKN45, included in each membrane, was determined to be 100 and intensities of the rest of the specimen were expressed in relation to the positive control.

*Staging and histological classification*

Staging was performed in accordance with The General Rules for TNM classification (International Union Against Cancer). Tissue specimens were promptly fixed in 10% formaldehyde, embedded in paraffin, and stained with hematoxylin and eosin. Tumour differentiation and degree of invasion were examined by the pathologists and histopathological classification was performed according to The General Rules for the Gastric Cancer Study (Japanese Research Society for Gastric Cancer, Kanehara Shuppan, Tokyo, Japan), and The General Rules for Clinical and Pathological Studies on Cancer of Colon, Rectum and Anus (Japanese Research Society for Cancer of Colon and Rectum, Kanehara Shuppan, Tokyo, Japan).

*Plasma CEA values*

The plasma CEA values of the patients were evaluated by a commercial assay kit (Wako Pure Chemical Industries, Osaka, Japan, cut-off value; 5 ng $ml^{-1}$) a few days prior to the operation. The clinical stages, histopathological classifications and plasma CEA values are summarised in Tables I and II.

*Statistics*

The Mann-Whitney test was performed for comparison of the non-parametrically distributed mRNA levels between different groups of specimen. Pearson's correlation coefficient was calculated between the mRNA levels and plasma values of CEA. Matched Wilcoxon test was performed for the comparison of mRNA levels between the metastatic tissues and their matching primary lesions.

### Results

*CEA and NCA mRNA levels in gastric and colon cancer tissues and normal mucosae*

The Northern blot analysis using the CEA and NCA cDNA probes revealed bands corresponding to CEA at 4.2 and 3.5-kb and a band corresponding to NCA at 2.9-kb in almost all specimens of gastric and colorectal malignancies (Figure 2 and 3). The number beneath each lane corresponds to the case number in Tables I and II.

There was, however, a significant difference (U = 53.5, $P<0.0005$) between the CEA mRNA levels in adenocarcinomas of stomach ($n = 27$) and colon ($n = 14$) as quantitated by the imaging analyser (Figure 4); the former being weaker than the latter. No significant difference in NCA gene expression on the other hand, was detected between the two groups (U = 95, $P>0.1$).

Although the mRNA levels of CEA in gastric cancer specimens ($n = 27$) were apt to be variable as discussed later, they were significantly (U = 111, $P<0.0005$) elevated compared with the mRNA levels in normal gastric mucosae ($n = 22$). CEA mRNA expression was, however, weakly but distinctly detected in some of the morphologically normal gastric mucosa. CEA gene expression was almost invariably high in normal colon mucosae ($n = 11$), and no statistically significant difference (U = 61, $P = 0.4$) was found between these and the mRNA levels in colorectal cancer specimens ($n = 14$).

NCA expression (Figure 4) proved to be more specific to cancerous tissues than that of CEA. It was detected in most colon cancer specimens, and there was a significant difference (U = 22.5, $P<0.05$) in the mRNA levels between colorectal cancer ($n = 12$) and normal colon mucosae ($n = 10$). The NCA expression in the normal gastric mucosae ($n = 20$) was also significantly weaker (U = 26, $P<0.0005$) than the gastric cancer tissues ($n = 26$), often too weak to be detected.

*CEA gene expression and plasma CEA value*

There was a lack of correlation between the gene expression in malignant tissues and the plasma CEA values both in gastric ($r = -0.056$, $P = 0.80$) and colorectal ($r = -0.021$, $P = 0.94$) cancer.

*CEA gene expression and metastasis*

In gastric cancer, no significant overexpression of CEA was observed (U = 27.5, $P = 0.37$ between pN0 and pN1, and U = 34, $P = 0.29$ between pN0 and pN2) between cancer tissues with clinically detectable nodal metastasis (pN1:$n = 6$, and pN2:$n = 14$) and those without (pN0:$n = 7$). The same result (U = 68, $P = 0.70$) came from comparison between gastric cancer tissues with distant metastasis ($n = 8$) and those without ($n = 19$).

In colon cancer, too, the comparison of CEA gene expression between node positive ($n = 6$) and node negative ($n = 8$) cancer tissues (U = *26*, $P = 0.75$), and cancer tissues with distant metastasis ($n = 2$) and those without ($n = 12$, U = 7,

**Figure 2** Northern blot analyses of CEA and NCA in surgical specimen from the patients with gastric cancer. Hybridisation with CEA and cDNA results in three bands; the bands at 4.2 Kb and 3.5 Kb correspond to CEA while the band at 2.9 Kb is the result of cross-hybridisation with NCA. Some of the bands not detected by exposure of 5-6 h appear by longer exposure. Hybridisation with NCA cDNA specifically detects NCA transcript at 2.9 Kb. Beta actin was used as an internal control. The case numbers correspond to the numbers in Table I. N stands for normal gastric mucosa, Ca for gastric cancer tissue, L for nodal metastasis, and H for liver metastasis, respectively.

**Figure 3** Northern blot analyses of CEA and NCA in surgical specimens from patients with colorectal cancer. The bands detected are identical to those described in the legend of Figure 2. The case numbers correspond to the numbers in Table II. The abbreviations are similar to those in Figure 2.

$P = 0.44$) resulted in no difference of statistical significance.

There was no significant difference either in NCA gene expression between cancer tissues with nodal or distant metastasis and those without both in gastric and colon cancers.

CEA mRNA levels in some metastatic tissues have also been quantitated (Tables I and II). The matched Wilcoxon test was performed between CEA gene expression in metastatic tissues and their matching primary lesions ($n = 9$). No significant overexpression of the CEA gene was detected ($P = 0.71$) in the metastatic tissues.

*CEA and NCA mRNA levels and histological classification*

There was an interesting correlation between CEA gene expression and pathological degree of differentiation in gastric cancer specimens (Figure 5). There was a tendency for well

**Figure 4** Left: The expression of CEA mRNAs in gastric mucosae ($n = 22$), gastric cancer specimen ($n = 27$), colorectal mucosae ($n = 11$), and colorectal cancer specimen ($n = 14$). The intensity of the major 3.5 Kb band of the cell line MKN45, determined by densitometric analysis with Fujix BAS2000 Imaging Analyzer was designated as 100, and the expression of the rest of the specimens were expressed in relation to MKN45. The bars denote mean + s.d. Right: The expression of NCA mRNAs in gastric mucosae ($n = 20$), gastric cancer tissues ($n = 26$), colorectal mucosae ($n = 10$), and colorectal cancer specimens ($n = 12$). The intensity of the 2.9 Kb transcript of the cell line MKN45 was designated as 100. The bars denote mean + s.d.

**Figure 5** The CEA and NCA mRNA levels (relative to those in MKN45 as described in the legend of Figures 4 and 5) of the gastric cancer tissues are shown in relation to the histopathological classification. The abbreviation *por* stands for poorly differentiated adenocarcinoma ($n = 7$), *mod* for moderately differentiated adenocarcinoma ($n = 11$) and *well* for well differentiated adenocarcinoma ($n = 4$), respectively.

and moderately differentiated gastric cancers ($n = 15$) to exceed the poorly differentiated cancers ($n = 8$) in CEA gene expression although the probability was calculated to be of borderline statistical significance ($U = 35, P = 0.055$). A significant difference ($U = 23, P = 0.024$), though, was observed between the NCA gene expression of well or moderately differentiated cancers ($n = 14$) and poorly differentiated cancers ($n = 8$).

### Discussion

CEA, one of the most widely used of the tumour markers in the diagnosis of colon cancer, has also been used in the management of gastric cancer. However, although the gene expression of the CEA-related gene family has been extensively examined using clinical samples of colon and lung cancer, gene expression in gastric cancer and gastric mucosa has not yet been evaluated in detail.

In our series of Northern blot analyses, CEA gene expression of varied levels has been recognised in gastric cancer tissues. Expression was significantly weaker compared with the almost invariable overexpression found in colon cancer, but it still exceeded the mRNA levels in normal gastric mucosae which were faint in most and not detected in seven cases. However, distinct expression of CEA mRNA was evident in some samples of the normal mucosae, including that of case 8, a morphologically normal mucosa derived from a patient with an early gastric cancer.

The mRNA levels of NCA in colon cancer have been reported to be significantly elevated relative to the normal mucosa (Chi, 1991) but the same results are now obtained with gastric cancer. Such altered expression implicates NCA as a more tumour-specific, if less organ-specific, tumour marker, provided the NCA specific antibody not cross-reacting with CEA is made available for the assay system.

On evaluating the contribution of CEA overexpression to metastasis, only the concomittant metastases found before or at the time of operation were taken into consideration in this study. Further periods of observation might allow the growth and detection as recurrences of micrometastases in the patients that we now diagnose as metastasis-free, and the results could then be different. At the moment, however, CEA was not found to be overexpressed in tumours with nodal or distant metastases compared with those without. Results such as those of case 3 or 11 in gastric cancer cast doubts to the role of CEA in some cases of metastasis formation, in spite of the encouraging *in vivo* study described earlier (Hostetter, 1988).

The total lack of correlation between the plasma CEA and CEA gene expression deserves some considerations. The plasma value is known to correlate with the pattern of immunostaining performed with the CEA monoclonal antibody in colon cancer specimens (Hamada, 1985). Normal colon mucosa exhibits an apical pattern of staining with low plasma CEA values while moderately differentiated carcinomas exhibit a cytoplasmic pattern, sometimes with distribution of CEA in the stroma, and result in high plasma CEA values. Our study, however, demonstrated abundant CEA gene expression in normal colon mucosa that is not significantly lower compared with the expression in cancer tissue. Nor was the CEA gene expression in the metastatic tissue strikingly overexpressed compared with the primary lesion. The overwhelming amount of normal colon mucosa in relation to the volume of cancerous tissue even in patients with large tumours and evidence of metastasis, then, makes it difficult to account for the 10- to 1000-fold elevation in serum CEA values as observed in some patients, solely from the point of view of the CEA gene expression. Some post-transcriptional mechanism as suggested by the pattern of immunostaining could underlie the mechanism of serum CEA elevation.

Cellular differentiation induced by sodium butyrate (Toribara, 1989) and gamma-interferon (Hauck, 1991), was reported to enhance CEA and NCA mRNA levels in human colon cancer cell lines. The levels of CEA released from the apical membranes into the medium rose in time-dependent manner when a colon cancer cell line, HT29-D4 was induced to differentiate by substituting galactose for glucose (Fantini, 1989). Whether the induction of CEA and NCA is the cause or the result of the differentiation is unknown, but our results concerning the expression of the CEA family and histopathological classification of gastric cancer samples seem to coincide with these studies. Gastric cancers often exhibit a chaotic mixture of portions with different degrees of differentiation, and pathologists are required to select the tissue type which they find is predominant on which to base their histopathological diagnosis. This perhaps is one of the reasons for the relatively large variation in mRNA expression among specimens of the same histopathological diagnosis. Significant differences in expression of NCA mRNA were nevertheless observed between poorly and well or moderately differentiated adenocarcinomas. One of the reasons for the almost invariable detection of CEA mRNA in colon cancer might then be that poorly differentiated adenocarcinomas are relatively rare in colon, moderately to well differentiated cancers being the predominant histological types found in colorectal region. The only poorly differentiated colon cancer among our specimen (case 3), indeed, was shown to express a relatively low CEA mRNA level.

It is interesting to note that the expression of another much investigated adhesion molecule, E-cadherin, is known to correlate with differentiation in squamous cell carcinoma of head and neck (Schipper, 1991) although the correlation is less clear in gastric cancer (Shimoyama, 1991). Further studies, though, will be needed to assess whether the CEA family, too, contributes to differentiation through its function as an adhesion molecule.

In conclusion, we postulate that (1) CEA mRNA level in gastric cancer is detectable but significantly lower than that of colon cancer, (2) the NCA mRNA is consistently overexpressed relative to normal mucosa both in gastric and colon cancer, (3) gene expression of CEA in cancer tissue does not correlate directly with serum CEA value in a patient and (4) association of the CEA family with differentiation has been demonstrated in gastric cancer specimens, although further studies will be needed to elucidate the true mechanism underlying this phenomenon.

We thank DR Hiroyuki Suenaga (Komaki, Japan) and Dr Hideyuki Katoh (Nakatsugawa, Japan) for kindly supplying some of the clinical samples, and Dr Akiko Tamakoshi (Department of Preventive Medicine, Nagoya University School of Medicine) for her help in the statistical analyses.

### References

BEAUCHEMIN, N., BENCHIMOL, S., COURNOYER, D., FUKS, A. & STANNERS, C.P. (1987). Isolation and characterization of full-length functional cDNA clones for human carcinoembryonic antigen. *Mol. Cell Biol.*, **7**, 3221–3230.

BENCHIMOL, S., FUKS, A., JOTHY, S., BEAUCHEMIN, N., SHIROTA, K. & STANNERS, C.P. (1989). Carcinoembryonic antigen, a human tumor marker, functions as an intercellular adhesion molecule. *Cell*, **57**, 327–334.

CHI, K., JESSUP, J.M. & FRAZIER, M.L. (1991). Predominant Expression of mRNA Coding for Nonspecific Cross-Reacting Antigen in Colorectal Carcinomas. *Tumor Biol.*, **12**, 298–308.

CHOMCZYNSKI, P. & SACCHI, N. (1987). Single-step method of RNA isolation by acid guanidium thiocyanate-phenol-chloroform extraction. *Anal. Biochem.*, **162**, 156–159.

COURNOYER, D., BEAUCHEMIN, N., BOUCHER, D., BENCHIMOL, S., FUKS, A. & STANNERS, C.P. (1988). Transcription of genes of the carcinoembryonic antigen family in malignant and nonmalignant human tissues. *Cancer Res.*, **48**, 3153–3157.

FANTINI, J., ROGNONI, J.-B., CULOUSCOU, J.-M., POMMIER, G., MARVALDI, J. & TIRARD, A. (1989). Induction of polarized apical expression and vectorial release of carcinoembryonic antigen (CEA) during the process of differentiation of HT29-D4 cells. *J. Cell Physiol.*, **141**, 126–134.

GOLD, P. & FREEDMAN, S.O. (1965). Demonstration of tumor-specific antigens in human colonic carcinoma by immunological tolerance and absorption techniques. *J. Exp. Med.*, **121**, 439–462.

HAMADA, Y., YAMAMURA, M., HIOKI, K., YAMAMOTO, M., NAGURA, H. & WATANABE, K. (1985). Immunohistochemical study of carcinoembryonic antigen in patients with colorectal cancer. *Cancer*, **55**, 136–141.

HASEGAWA, T., ISOBE, K., TSUCHIYA, Y., OIKAWA, S., NAKAZATO, H., NAKASHIMA, I. & SHIMOKATA, K. (1993). Nonspecific cross-reacting antigen (NCA) is a major member of the carcinoembryonic antigen (CEA)-related gene family expressed in lung cancer. *Br. J. Cancer*, **67**, 58–65.

HAUCK, W. & STANNERS, C. (1991). Control of carcinoembryonic antigen family expression in a differentiating colon carcinoma cell line, Caco-2. *Cancer Res.*, **51**, 3526–3533.

HIGASHIDE, T., HINODA, Y., ITOH, J., TAKAHASHI, H., SATOH, Y., IBAYASHI, Y., IMAI, K. & YACHI, A. (1990). Detection of mRNAs of carcinoembryonic antigen and nonspecific cross-reacting antigen genes in colorectal adenomas and carcinomas by *in situ* hybridization. *Jpn. J. Cancer Res.*, **81**, 1149–1154.

HINODA, Y., TAKAHASHI, H., HIGASHIDE, T., NAKANO, T., ARIMURA, Y., YOSHIMOTO, M., TSUIJISAKI, M., IMAI, K. & YACHI, A. (1991). Correlated expression of mRNAs of carcinoembryonic antigen and nonspecific cross-reacting antigen genes in malignant and nonmalignant tissues of the colon. *Jpn. J. Clin. Oncol.*, **21**, 75–81.

HOSTETTER, R.B., AUGUSTUS, L.B., MANKARIOUS, R., CHI, K., FAN, D., TOTH, C., THOMAS, P. & JESSUP, J.M . (1990). Carcinoembryonic antigen as a selective enhancer of colorectal cancer metastasis. *J. Natl Cancer Inst.*, **82**, 380–385.

KAMARCK, M.E., ELTING, J.J., HART, J.T., GOEBEL, S.J., RAE, P.M.M., NOTHDURFT, M.A., NEDWIN, J.J. & BARNETT, T.R. (1987). Carcinoembryonic antigen family: expression in a mouse L-cell transfectant and characterization of a partial cDNA in bacteriophage gt11. *Proc. Natl Acad. Sci. USA*, **84**, 5350–5354.

KUROKI, M., KOGA, Y. & MATSUOKA, Y. (1981). Purification and characterization of carcinoembryonic antigen-related antigens in normal adult feces. *Cancer Res.*, **41**, 713–720.

LIOTTA, L.A. & STETLER-STEVENSON, W.G. (1991). Tumor invasion and metastasis: An imbalance of positive and negative regulation. *Cancer Res.*, **51**, 5054s–5059s.

NAKAJIMA-IIJIMA, S., HAMADA, H., REDDY, P. & KAKUNAGA, T. (1985). Molecular structure of the human cytoplasmic beta-actin gene: interspecies homology of sequences in the introns. *Proc. Natl Acad. Sci. USA*, **82**, 6133–6137.

NEUMAIER, M., ZIMMERMANN, W., SHIVELY, L., HINODA, Y., RIGGS, A.D. & SHIVELY, J.E. (1988). Characterization of a cDNA clone for the nonspecific cross-reacting antigen (NCA) and a comparison of NCA and carcinoembryonic antigen. *J. Biol. Chem.*, **263**, 3202–3207.

OIKAWA, S., NAKAZATO, H. & KOSAKI, G. (1987). Primary structure of human carcinoembryonic antigen (CEA) deduced from cDNA sequence. *Biochem. Biophys. Res. Commun.*, **142**, 511–518.

SATO, C., MIYAKI, M., OIKAWA, S., NAKAZATO, H. & KOSAKI, G. (1988). Differential expression of carcinoembryonic antigen and nonspecific crossreacting antigen genes in human colon adenocarcinomas and normal colon mucosa. *Jpn. J. Cancer Res.*, **79**, 433–437.

SCHIPPER, J.H., FRIXEN, U.H., BEHRENS, J., UNGER, A., JAHNKE, K. & BIRCHMEIER, W. (1991). E-cadherin expression in squamous cell carcinoma of head and neck: Inverse correlation with tumor dedifferentiation and lymph node metastasis. *Cancer Res.*, **51**, 6328–6337.

SHIMOYAMA, Y. & HIROHASHI, S. (1991). Expression of E- and P-cadherin in gastric carcinomas. *Cancer Res.*, **51**, 2185–2192.

TAWARAGI, Y., OIKAWA, S., MATSUOKA, Y., KOSAKI, G. & NAKAZATO, H. (1988). Primary structure of nonspecific cross-reacting antigen (NCA), a member of carcinoembryonic antigen (CEA) gene family, deduced from cDNA squence. *Biochem. Biophys. Res. Commun.*, **150**, 89–96.

TORIBARA, N.W., SACK, T.L., GUM, J.R., HO, S.B., SHIVELY, J.E., WILLSON, J.K.V. & KIM, Y.S. (1989). Hererogeneity in the induction and expression of carcinoembryonic antigen-related antigens in human colon cancer cell lines. *Cancer Res.*, **49**, 3321–3327.

ZIMMERMAN, W., ORTLIEB, B., FRIEDRICH, R. & VON KLEIST, S. (1987). Isolation and characterization of cDNA clones encoding the human carcinoembryonic antigen reveal a highly conserved repeating structure. *Proc. Natl Acad. Sci. USA*, **84**, 2960–2964.